# Stressful Life Events, Unhealthy Eating Behaviors and Obesity among Chinese Government Employees: A Follow-Up Study

**DOI:** 10.3390/nu15112637

**Published:** 2023-06-05

**Authors:** Dan Qiu, Jun He, Yilu Li, Feiyun Ouyang, Shuiyuan Xiao

**Affiliations:** 1 Department of Social Medicine and Health Management, Xiangya School of Public Health, Central South University, Changsha 410078, China; qiudan@csu.edu.cn (D.Q.);; 2 Department of Psychiatry, Yale School of Medicine, New Haven, CT 06510, USA; 3 Mental Health Institute, Second Xiangya Hospital, Central South University, Changsha 410078, China

**Keywords:** stress, unhealthy eating behavior, obesity, path analysis, bootstrap analysis

## Abstract

**Background:** The underlying mechanisms of the relationship between stressful life events and obesity among Chinese workers are unclear. **Objective:** This study aimed to understand the processes and mechanisms involved in stressful life events, unhealthy eating behavior, and obesity among Chinese workers. **Methods:** From January 2018 to December 2019, a total of 15,921 government employees were included at baseline and they were followed-up until May 2021. Stressful life events were assessed using the Life Events Scale, and unhealthy eating behavior was assessed using four items. BMI was calculated as weight (kg) divided by height (m^2^) using physically measured data. **Results:** Overeating at each mealtime (OR = 2.21, 95%CI: 1.78–2.71) at baseline led to reports of higher risk of obesity at follow up. Eating before going to bed at night sometimes (OR = 1.51, 95%CI: 1.31–1.73) or often (OR = 3.04, 95%CI: 2.28–4.05) at baseline led to reports of higher risk of obesity at follow-up. Eating out sometimes (OR = 1.74, 95%CI: 1.47–2.07) or often (OR = 1.59, 95%CI: 1.07–2.36) at baseline led to reports of higher risk of obesity at follow-up. Stressful life events were not directly associated with obesity, but unhealthy eating behaviors, including overeating at each mealtime (β = 0.010, 95%CI: 0.007–0.014; β = 0.002, 95%CI: 0.001–0.004, respectively) and irregular meal timing (β = −0.011, 95%CI: −0.015–−0.008; β = −0.004, 95%CI: −0.006–−0.001, respectively), significantly mediated the associations between stressful life events at baseline and obesity at both baseline and follow-up. **Conclusions:** Unhealthy eating behaviors mediated the relationship between stressful life events and obesity. Interventions should be provided to workers who have experienced stressful life events and unhealthy eating behaviors.

## 1. Introduction

Obesity is defined as a disorder and characterized by excess body fat, which can lead to health complications such as cardiovascular disease, hypertension, sleep disorders, and type-2 diabetes [1]. As a multifactorial problem, obesity has been prevalent in developed countries for many years, but it is becoming increasingly present in developing countries as well [2]. From 2010 to 2014, in the Western Pacific, the prevalence of overweight and obesity in adults increased from 5.30% to 6.90% [3].

Stressful life events have been reported in the course of many chronic diseases [4]. Previous studies have shown that stress is an important cause of the development of overweight and obesity [5]. Currently, obesity has become a concern among working adults due to prolonged life stressors [6,7,8]. Lots of individuals with obesity report having experienced a very stressful situation prior to the disease manifestation, and in some cases, describe ongoing exposure and other determinants [5,7]. It is said that perceived stress may influence people’s daily dietary practices, because it causes their long-term dietary patterns to deviate further from the recommended nutritional guidelines [5,9,10,11]. Although previous work has examined the relationship between stress and obesity, no longitudinal studies have examined how perceived stress may exacerbate or buffer this relationship in Chinese working adults. Understanding what kinds of eating behavior are selected under stress is a crucial issue both for theoretical interpretation of the mechanisms involved in and predicting the deleterious effects of perceived stress on people’s health [12]. Previous results reported that stress may promote irregular eating patterns and strengthen networks towards unhealthy eating behaviors (such as hedonic overeating), and these effects may be exacerbated in individuals with overweight and obesity. The factors underlying different behaviors that contribute to overweight and obesity are slowly becoming understood.

Over the past three decades, in parallel to the rapid increase in overweight or obesity across the world, people living in China have experienced drastic social, economic, and environmental transitions [13]. Lifestyle-related factors, such as dietary habits and physical activity, among the Chinese population have changed dramatically [14]. Currently, the data show that China has the highest number of people with overweight and obesity across the world [15]. People’s eating behaviors are shaped by complex factors and their interactions in the expanding obesogenic environment, food environment, dietary patterns, and lifestyles. The underlying mechanisms of the relationship between stressful life events and obesity among working adults in China are unclear. It is important to understand the processes and mechanisms involved in stressful life events, eating behaviors, and obesity among working adults. In this study, the levels of obesity in Chinese government employees and their associations with stressful life events were investigated first. We further explored the roles of unhealthy eating behaviors as mediators of such associations (associations between stressful life events and obesity) via fitting path analysis. To elucidate the relationships and the underlying mechanisms between stressful life events and obesity, a serial multiple mediation model was used in this follow-up study. Specifically, this study hypothesized that (1) stressful life events are associated with unhealthy eating behaviors; (2) unhealthy eating behaviors mediate the association between stressful life events and obesity.

## 2. Methods

### 2.1. Ethics Statement

This study was approved by the Ethics Committee of Xiangya School of Public Health, Central South University (Ethics approval number: XYGW-2016-10). Written informed consent was obtained prior to data collection from participants.

### 2.2. Study Design and Participants

The present study is based on a prospective longitudinal cohort study formed from a working population cohort in Southern China, designed to measure the relationships between behavior and subsequent health outcomes. This cohort study was conducted between January 2018 and May 2021, and was carried out in five cities of Hunan Province, China. Specially, the baseline survey was conducted from January 2018 to December 2019. The follow-up survey was conducted from January 2020 to December 2021. In each city, there was a general hospital that cooperated with this project as the places for health examinations and investigations to be conducted. The project aimed to investigate government employees who were aged 18–60 years old and working in Hunan province [16]. During the program, a digital self-reporting questionnaire platform was established to collect related information about these included employees. Recruited government employees accessed the questionnaire via URLs sent using a short messaging service and finished the questionnaire via cellphone, tablet, or PC. The same questionnaire was used for both the baseline and follow-up surveys. In the project, a total of 36 public sectors or organizations that agreed to answer the questionnaire and finish the health examinations were recruited. All government employees in the 36 included public sectors or organizations were invited to complete the required health examinations and relevant questionnaire. After written informed consent was obtained, government employees were included in the study and completed the relevant questionnaires. See Figure S1 for the details.

### 2.3. Measures

#### 2.3.1. Stressful Life Events

Stressful life events of Chinese government employees were assessed using the Life Events Scale (LES), the details of which were described in our previous study [17]. There are 48 items in this scale, which were classified into different dimensions, including stressful life events related to marriage and family, stressful life events related to work and study, health-related events, stressful life events related to accidents, legal disputes, and economic problems. Based on participants’ daily experiences over the past year, Chinese government employees were asked to choose “yes” or “no” for each item. In the current study, the Chinese version of the Life Events Scale was used, for which our previous study reported favorable validity and reliability and which is widely used in China [17]. In this study, we divided stressful life events into four groups according to the number of occurrences: none, one, two, and three or more stressful life events.

#### 2.3.2. Unhealthy Eating Behavior

Referring to previous studies [12,18,19], the daily eating habits of Chinese government employees were assessed using four items in this study. The four-item questionnaire assessed whether the employees had engaged in the following unhealthy eating behaviors over the past year: frequent irregular meal timing, overeating at each mealtime, eating out frequently, and eating before going to bed at night frequently. In this study, “unhealthy eating behaviors” were assessed via self-report, with response options including “not at all”, “sometimes”, and “often” on a three-point Likert-type scale.

Participants were asked in the four questions: (a) “generally, with what frequency have you experienced irregular meal timing over the past year?”; (b) “generally, with what frequency have you experienced overeating at each mealtime over the past year?”; (c) “generally, with what frequency have you experienced eating out over the past year?”; (d) “generally, with what frequency have you experienced eating before going to bed at night over the past year?”. Employees rated the items on a three-point Likert-type scale, with response options including not at all (less than once a week), sometimes (about once a week), and often (≥2 times a week), and the score ranged from 0~2. A higher score means more frequent unhealthy eating behavior.

#### 2.3.3. Obesity

At both baseline and follow-up, the height and weight of Chinese government employees were measured in the intensive assessment subsample by a trained doctor using a stadiometer and scale, respectively. BMI was calculated as weight (kg) divided by height (m^2^) using physically measured data. Based on the guidelines published in China, Chinese government employees with a BMI ranging from 24.00 kg/m^2^ to 27.90 kg/m^2^ were defined as overweight, and those with a BMI ≥ 28.00 kg/m^2^ were defined as obese [14].

#### 2.3.4. Covariates

Sociodemographic variables included gender, age, household income, education background (secondary school or below, university, or university or above), marital status (married, single, divorced, or widowed), grades of employment, working hours per day, type of work, self-reported illness (yes or no), and lack of psychical activity (yes or no). The details for these covariates were described in our previous study.

#### 2.3.5. Statistical Analysis

In this study, SPSS 26.0 was used for data analysis. Odds ratios (OR) and 95% confidence intervals (95% CI) were calculated to explore the associations between stressful life events, unhealthy eating behaviors, and obesity using logistic regression analysis. In the regression models, we adjusted for gender, age, family income, education level, marital status, grades of employment, working hours per day, type of work, whether the participant had a chronic illness, and psychical activity. All the *p*-values refer to two-tailed tests in this study, and a *p*-value < 0.05 was considered statistically significant.

To examine the correlations between stressful life events, unhealthy eating behaviors, and obesity, Pearson’s correlation analysis was conducted. To examine the goodness of fit of the underlying constructs in the proposed model [20], the measurement model in the current analysis was tested using confirmatory factor analysis (CFA). To test the hypothesized directionality of the associations between the constructs, and the overall fitness of the model, path analysis was conducted. Four indicators, including the result of the χ^2^ test, the comparative fit index (CFI), the non-normed fit index (NNFI), and the root mean square error of approximation (RMSEA), were calculated to test how well the model fitted the included data [20]. Based on our previous study, a value of RMSEA < 0.08, and a value of CFI and a value of NNFI greater than 0.85, indicate a reasonable model fit [16]. To identify the mediation effects of unhealthy eating behavior on the associations between stressful life events and obesity, a bootstrap procedure was conducted in the path analysis [20,21]. In the bootstrap analysis, bias-corrected 95% CIs based on 5000 resamples were calculated [21]. When the 95% CI did not include zero, a statistically significant mediation effect was observed.

## 3. Results

### 3.1. Characteristics of Included Participants

After excluding participants with missing demographic and obesity data, there were 15,921 employees at baseline and 12,621 employees at follow-up available for the current analysis. See Figure S1 for the details.

At baseline, the mean age of the included Chinese government employees was 37.82 ± 10.22 years old. Additionally, 60.82% of the Chinese government employees were female, 78.14% of them reported that they were married/living with a partner, 68.40% of them said that they had received a college education, and 56.41% had a mentally oriented job. Additionally, 48.02% of the included government employees reported that they lacked physical exercise, 11.68% of employees were current smokers, 10.01% of employees were current drinkers, and 63.18% reported that they usually worked 7–8 h per day. See Table 1 and Supplementary data for the details.

### 3.2. Associations between Stressful Life Events, Unfavorable Eating Behavior, and Obesity

In the adjusted model, government employees who overate at each mealtime (OR = 2.28, 95%CI: 1.90–2.74) sometimes at baseline had a higher risk of obesity at baseline. Government employees who ate before going to bed at night sometimes (OR = 1.34, 95%CI: 1.18–1.51) or often (OR = 1.63, 95%CI: 1.23–2.15) at baseline had a higher risk of obesity at baseline. Government employees who ate out sometimes (OR = 1.46, 95%CI: 1.24–1.71) or often (OR = 1.61, 95%CI: 1.14–2.27) at baseline reported had a higher risk of obesity at baseline.

Additionally, government employees who overate at each mealtime (OR = 2.21, 95%CI: 1.78–2.71) at baseline had a higher risk of obesity at follow up. Government employees who ate before going to bed at night sometimes (OR = 1.51, 95%CI: 1.31–1.73) or often (OR = 3.04, 95%CI: 2.28–4.05) at baseline had a higher risk of obesity at follow-up. Government employees who ate out sometimes (OR = 1.74, 95%CI: 1.47–2.07) or often (OR = 1.59, 95%CI: 1.07–2.36) at baseline had a higher risk of obesity at follow-up.

Government employees who experienced irregular meal timing often (OR = 2.28, 95%CI: 1.90–2.74) at baseline had a higher risk of obesity at follow-up. However, the associations between stressful life events and obesity were not significant in the regression models. See Table 2 for the details.

### 3.3. Correlations among the Variables

The results of the Pearson’s correlation analysis showed that stressful life events at baseline were significantly correlated with irregular meal timing, overeating at each mealtime, eating before going to bed at night, and eating out at baseline (r = 0.19, 0.17, 0.08, and −0.02, respectively; *p* < 0.05). Stressful life events at baseline were significantly correlated with obesity at baseline and follow-up (r = −0.04 and −0.05, *p* < 0.05). Irregular meal timing, overeating at each mealtime, eating before going to bed at night, and eating out at baseline were significantly correlated with obesity at baseline (r = −0.04, 0.02, 0.08, and 0.11, respectively; *p* < 0.05) and obesity at follow-up, respectively (r = −0.04, 0.03, 0.08, and 0.12, respectively; *p* < 0.05). Additionally, irregular meal timing was significantly correlated with overeating at each mealtime, eating before going to bed at night, and eating out at baseline (r = 0.31, 0.17, and 0.07, respectively; *p* < 0.05). Eating before going to bed at night was significantly correlated with overeating at each mealtime and eating out at baseline (r = 0.18 and 0.15, *p* < 0.05). Overeating at each mealtime was significantly correlated with eating out at baseline (r = 0.16, *p* < 0.05). See Table 3 for the details.

### 3.4. Model Testing and Path Coefficients

**Figure 1:**
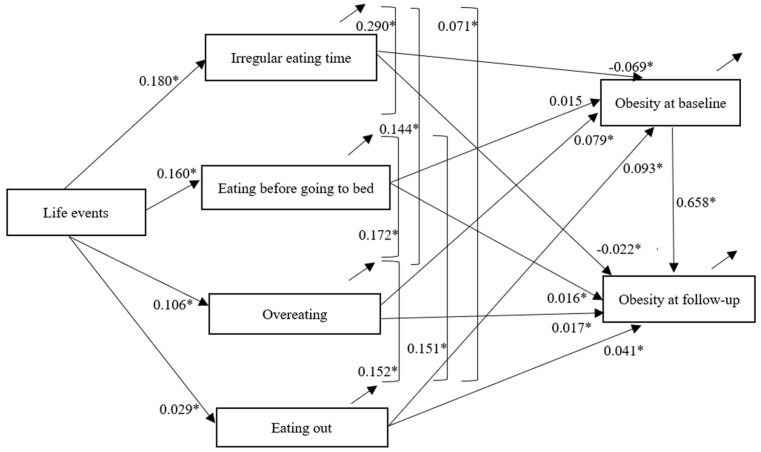
The proposed path model with standardized coefficients. For simplicity reasons, the background variables controlled in this model are not shown in the figure. Note: * *p* < 0.05.

Validation analysis of the structural equation model indicated that the model fits the data well (Figure 1), χ^2^(2) = 7.563, *p* = 0.023, CFI = 0.99, NNFI = 0.99, RMSEA = 0.01. All factor loadings were significant at *p* < 0.001.

As presented in Figure 1, at baseline, the direct path from stressful life events to irregular meal timing (B = 0.18, β = 0.04, *p* < 0.001), overeating at each mealtime (B = 0.11, β = 0.01, *p* < 0.001), eating before going to bed at night (B = 0.16, β = 0.03, *p* < 0.001) and eating out (B = 0.01, β = 0.03, *p* < 0.001) was significant and positive, respectively. The direct path from irregular meal timing (B = −0.50, β = −0.07, *p* < 0.001) to obesity was significant and negative, and the direct path from overeating at each mealtime (B = 1.67, β = 0.08, *p* < 0.001), and eating out at baseline (B = 1.15, β = 0.09, *p* < 0.001) to obesity at baseline was significant and positive, respectively. However, the direct path from eating before going to bed at night (B = −0.13, β = 0.02, *p* = 0.120) to obesity at baseline was not significant.

At follow-up, the direct path from irregular meal timing (B = −0.17, β = −0.02, *p* = 0.002) at baseline to obesity at follow-up was significant and negative, and the direct path from eating before going to bed at night (B = 0.14, β = 0.02, *p* = 0.028), overeating at each mealtime (B = 0.37, β = 0.02, *p* = 0.013), and eating out at baseline (B = 0.53, β = 0.04, *p* < 0.001) to obesity at follow-up was significant and positive, respectively.

In addition, a higher prevalence of irregular meal timing was associated with a higher prevalence of overeating at each mealtime, eating before going to bed at night, and eating out. A higher prevalence of eating before going to bed at night was associated with a higher prevalence of overeating at each mealtime. A higher prevalence of overeating at each mealtime was associated with a higher prevalence of eating out. A higher prevalence of obesity at baseline was associated with a higher prevalence of obesity at follow up.

### 3.5. Mediation Effects of Unhealthy Eating Behavior between Stressful Life Events and Obesity

Bootstrapping analyses indicated that stressful life events at baseline were indirectly associated with obesity at both baseline and follow-up through irregular meal timing (β = −0.012 and −0.004, *p* < 0.05). Additionally, the individual mediation effect of overeating at each mealtime for the associations between stressful life events at baseline and obesity at both baseline and follow-up was statistically significant (β = 0.008 and 0.002, *p* < 0.05). Stressful life events at baseline were indirectly associated with obesity at both baseline and follow-up through eating out frequently (β = 0.003 and 0.001, *p* < 0.05). Additionally, the individual mediation effect of eating before going to bed for the associations between stressful life events at baseline and obesity at baseline was not statistically significant (β = 0.002, *p* > 0.05), but was significant for the associations between stressful life events at baseline and obesity at follow-up (β = 0.001, *p* < 0.05). See Table 4 for the details.

Furthermore, stressful life events at baseline were indirectly associated with obesity at follow-up through irregular meal timing (β = −0.008, *p* < 0.01) and obesity at baseline. Additionally, the individual mediation effect of overeating at each mealtime and obesity at baseline for the associations between stressful life events at baseline and obesity at follow-up was statistically significant (β = 0.06, *p* < 0.01). Stressful life events at baseline were indirectly associated with obesity at follow-up through eating out frequently (β = 0.002, *p* < 0.05) and obesity at baseline. However, the individual mediation effect of eating before going to bed and obesity at baseline for the associations between stressful life events at baseline and obesity at follow-up was not statistically significant (β = 0.002, *p* > 0.05). See Table 4 for the details.

## 4. Discussion

### Comparison with Previous Research

Previous studies have reported on the adverse effects of stressful life events on obesity [6,8,9]. In the current study, we found that stressful life events were not directly associated with obesity in Chinese government employees, but unhealthy eating behaviors, including overeating at each mealtime, eating out, and irregular meal timing, significantly mediated the relationships between stressful life events at baseline and obesity at both baseline and follow-up, while eating before going to bed at night significantly mediated the associations between stressful life events at baseline and obesity at follow-up, which was consistent with previous studies [22,23,24,25,26].

Based on Lazarus’s psychological stress and coping theory, individuals who experience stress often perceive that they do not have enough resources to handle the demands of their environment [27]. Thus, the associations between perceived stress and eating behaviors may depend upon people’s perceived ability to effectively manage the stress that they have experienced (hereafter referred to as perceived management) [18]. If unhealthy eating behavior is used as means to regulate emotional experiences, one would expect that the extent to which stressful life events alter health behaviors is influenced by one’s emotional reactions to the events. Some researchers have found that stress-related negative affect is associated with hedonic overeating. Thus, Chinese employees who experience stressful life events may perceive an inability to regulate their emotional experiences, and then, unhealthy eating behaviors occur and lead to obesity. The long-term activation model suggests that, next to the stressor itself, perceived cognitive anticipation of the stressor may lead to stress-related physiological activity and insufficient physical recovery, and result in poor health [28]. It is noted that there are three forms of cognitive anticipation, including positive cognitive anticipation, negative cognitive anticipation, and positive outcome expectancy [29]. It is possible that Chinese government employees who focus on their work in a negative way (such as negative cognitive anticipation of a stressful work environment) are likely to experience a negative impact on obesity [30]. Thus it is important to identify employees who have experienced stressful life events and provide stress management for companies or organizations across the world [31].

Specifically, stressful life events were indirectly associated with obesity through irregular meal timing. A higher prevalence of irregular meal timing was associated with a lower prevalence of obesity among Chinese government employees. It is said that the classification of meal time distribution is usually based on cultural norms, such as the division into breakfast, lunch, and dinner, which reflects not only the meal time but also the size of the eating occasion [32]. Chinese government employees who experience irregular meal timing under stress may experience a negative impact on energy balance, which may facilitate weight loss [32]. In turn, Chinese government employees who experience overeating at each meal time under stress may experience higher energy intake, which can lead to obesity [33]. As expected, the results of path analysis showed that Chinese government employees who experienced stressful life events may exhibit a higher prevalence of eating out, and eating out could be associated with higher energy intake, poor dietary quality, or an increased risk of weight gain and obesity [34,35]. In addition, previous studies have shown that obese adults perceive eating out as a key barrier to sustained adherence to a specific dietary regime [36]. Therefore, modifying this parameter may be a reasonable strategy to promote healthy eating patterns and prevent weight gain [37]. However, in order to build a scientific case for action and develop appropriate public health strategies, a comprehensive understanding of the specifics of eating out and how they relate to diet quality is needed in the future [35]. Additionally, Chinese government employees who experienced stressful life events exhibited a higher prevalence of eating before going to bed at night, and eating before going to bed at night was associated with an increased risk of weight gain and obesity. This result was consistent with previous studies, in which the timing of dietary activity has been considered a factor in the multifactorial pathology of obesity, and eating at night may be deleterious for humans because it is opposite to the normal day/night cycle [38]. A disturbed day/night cycle increases the risk of developing obesity [26].

## 5. Future Implications

Firstly, the current obesity prevention strategies across the world focus mainly on eating behaviors and physical activity. The results of the current study suggest that stress is a quite important but overlooked public policy target. Given that many government employees have experienced a variety of stressful life events and the negative effects of those stressful life events on obesity are significant, it is important to explore the boundary management between personal life and work among Chinese employees [39], which may contribute to better health outcomes [40]. Secondly, Yau et al. found that obesity itself can represent a state of stress due to the high prevalence of weight stigma, and they suggest that there is a vicious cycle of stress to obesity to weight stigma to stress [12]. Future studies could explore the contribution of weight stigma to stress and obesogenic processes in Chinese occupational populations [12]. Conducting health management in the workplace is shown to have improved employees’ work performance, but the protocol for these health management processes among employees is still under development, with no available programs yet for government employees. This needs to be verified with scientific evidence in the future [41]. A previous study found that strategies to improve the implementation of workplace-based interventions may be effective if the factors that impede implementation can be addressed [42]. However, the determinants of program implementation are usually complex [43]. In addition, considering that Chinese workers are generally hard-working and compliant due to their social culture, we think the negative impact of work-related stress is particularly high in Chinese employees, especially for those working in government. In summary, workplace-based health management for Chinese government employees based on scientific evidence should be develop in the future. Finally, previous studies have reported that stress could trigger physiological changes in the hypothalamic–pituitary–adrenal axis of individuals, reward processing in their brain, and gut microbial changes [44]. Although this study controlled for many potential confounders in the model, further exploration of the biological mechanisms influencing the association between stress and obesity is pending. In addition, during the follow-up period, the outbreak of COVID-19 occurred. Whether the occurrence of the COVID-19 pandemic and the isolation policy associated with COVID-19 changed workers’ eating behaviors and related health outcomes requires further future exploration.

## 6. Limitations

The present study has some limitations. Firstly, a previous study indicated that different people may feel different about the stressors they have experienced [4]. Thus, stressful life events should be classified into different types according to their reporting [4]. It is necessary to ask these working adults to further evaluate whether the events they have experienced are positive stressful life events or negative stressful life events in the future, which may help us gain a better understanding of the associations between stressful life events, unhealthy eating behavior, and obesity. Secondly, it is possible that the assessment of unhealthy eating behavior in this study could not accurately capture the multi-dimensional nature of eating behaviors among Chinese working adults. Thus, we cannot completely exclude misclassification bias as a source of error that explains the observed associations in the current study. Thirdly, this study was conducted in Chinese workers, so the conclusions drawn need to be extrapolated with caution to other races or other regions. Finally, although this study has adjusted for many potential confounders in the mediation models, the results might still be affected by unmeasured confounding factors (such as the outbreak of COVID-19, genetic background, as well as coping style).

## 7. Conclusions

Government employees who overate at each mealtime, ate before going to bed at night, and ate out at baseline had a higher risk of obesity at follow up. However, the associations between stressful life events and obesity were not significant. The results of path analysis showed that stressful life events were not directly associated with obesity, but unhealthy eating behaviors, including overeating at each mealtime and irregular meal timing, significantly mediated the associations between stressful life events at baseline and obesity at both baseline and follow-up, while eating before going to bed at night significantly mediated the associations between stressful life events at baseline and obesity at follow-up.

## Figures and Tables

**Table 1:** Demographic characteristics among Chinese government employees.

Variables	Baseline (*n* = 15,921)		Follow-Up (*n* = 12,621)	
Demographic Characteristics	n	%	n	%
Gender				
Male	6238	39.18	4296	26.98
Female	9683	60.82	8325	52.29
Age(year)				
18–30	4794	30.11	3443	21.63
31–40	5744	36.08	4688	29.45
41–50	3491	21.93	2852	17.91
51–60	1892	11.88	1638	10.29
Education				
High school or below	941	5.91	541	3.40
University	10,890	68.40	8999	56.52
Graduate	4090	25.69	3081	19.35
Marital status				
Single	3108	19.52	2074	13.03
Married	12,440	78.14	10,213	64.15
Divorced/widowed	373	2.34	334	2.10
Family annual income (RMB)				
≤10,000	7076	44.44	5138	32.27
100,000–300,000	7875	49.46	6759	42.45
≥310,000	970	6.09	724	4.55
Presence of comorbidity				
Yes	2377	14.93	2757	17.32
No	13,829	86.86	9885	62.09
Type of work				
Mentally oriented	8981	56.41	6602	41.47
Physically oriented	6940	43.59	6019	37.81
Grades of employment				
Primary	7624	47.89	6207	38.99
Intermediate	5505	34.58	4100	25.75
Senior/deputy senior	2792	17.54	2314	14.53
Working hours per day				
<6 h	1308	8.22	999	6.27
6–8 h	10,059	63.18	8007	50.29
>8 h	4554	28.60	3615	22.71
Drinking				
Yes	1594	10.01	1014	6.37
No	14,327	89.99	11,607	72.90
Smoking				
Yes	1859	11.68	1105	6.94
No	14,062	88.32	11,506	72.27
Lack of exercise				
Yes	7645	48.02	6192	38.89
No	8276	51.98	6429	40.38
Self-reported chronic disease				
Yes	2352	14.77	2756	17.31
No	13,569	85.23	9865	61.96
Number of life events				
0	4810	30.21	3845	24.15
1	2599	16.32	2017	12.67
2	2256	14.17	1741	10.94
≥3	6541	41.08	5018	31.52
BMI				
<18.5	9874	62.02	662	4.16
18.5–23.9	9385	58.95	7231	45.42
24–27.9	4447	27.93	3475	21.83
≥28	1500	9.42	1293	8.12

**Table 2:** Odds ratios of obesity for stressful life events and unfavorable eating behavior.

	Obesity	
	At Baseline (*n* = 15,921)	At Follow-Up (*n* = 12,621)
Stressful life events		
0	1	1
1	0.91 (0.76–1.09)	0.79 (0.66–1.00)
2	1.02 (0.85–1.22)	0.82 (0.67–1.01)
≥3	1.05 (0.89–1.21)	0.88 (0.75–1.01)
Irregular meal timing		
No	1	1
Sometimes	1.08 (0.89–1.32)	1.02 (0.88–1.16)
Often	0.97 (0.86–1.10)	0.72 * (0.57–0.91)
Overeating at each mealtime		
No	1	1
Sometimes	2.28 * (1.90–2.74)	2.21 * (1.78–2.71)
Often	-	-
Eating before going to bed at night		
No	1	1
Sometimes	1.34 * (1.18–1.51)	1.51 * (1.31–1.73)
Often	1.63 * (1.23–2.15)	3.04 * (2.28–4.05)
Eating out		
No	1	1
Sometimes	1.46 * (1.24–1.71)	1.74 * (1.47–2.07)
Often	1.61 * (1.14–2.27)	1.59 * (1.07–2.36)

* *p* < 0.05.

**Table 3:** The results of Pearson’s correlation.

Variables	(1)	(2)	(3)	(4)	(5)	(6)
(1) Stressful life events	1					
(2) Irregular meal timing	0.19 *	1				
(3) Eating before going to bed	0.17 *	0.31 *	1			
(4) Overeating at each mealtime	0.08 *	0.18 *	0.18 *	1		
(5) Eating out	−0.02 *	0.07 *	0.15 *	0.16 *	1	
(6) Obesity at baseline	−0.04 *	−0.04 *	0.02 *	0.08 *	0.11 *	1
(7) Obesity at follow-up	−0.05 *	−0.04 *	0.03 *	0.08 *	0.12 *	0.67 *

* *p* < 0.05.

**Table 4:** Bootstrap analyses of indirect effects of the mediation model.

Cross-Sectional Paths for Obesity at Baseline	β	Boot SE	95%CI ^a^	*p*
*Stressful life events → irregular meal timing → obesity at baseline*	−0.012	0.002	−0.015–−0.008	*p* < 0.01
*Stressful life events → eating before going to bed → obesity at baseline*	0.002	0.002	−0.002–0.004	*p* > 0.05
*Stressful life events → overeating at each mealtime → obesity at baseline*	0.008	0.002	0.007–0.014	*p* < 0.01
*Stressful life events → eating out → obesity at baseline*	0.003	0.002	0.001–0.008	*p* < 0.05
**Longitudinal paths for obesity at follow-up**				
*Stressful life events → irregular meal timing → obesity at follow-up*	−0.004	0.001	−0.006–−0.001	*p* < 0.05
*Stressful life events → eating before going to bed → obesity at follow-up*	0.002	0.001	0.001–0.004	*p* < 0.05
*Stressful life events → overeating at each mealtime → obesity at follow-up*	0.002	0.001	0.001–0.004	*p* < 0.05
*Stressful life events → eating out → obesity at baseline*	0.001	0.001	0.001–0.003	*p* < 0.05
**Longitudinal paths for obesity at baseline and follow-up**				
*Stressful life events → irregular meal timing → obesity at baseline → obesity at follow-up*	−0.008	0.001	−0.011–−0.006	*p* < 0.01
*Stressful life events → eating before going to bed → obesity at baseline → obesity at follow-up*	0.002	0.001	−0.001–0.004	*p* > 0.05
*Stressful life events → overeating at each mealtime → obesity at baseline → obesity at follow-up*	0.006	0.001	0.004–0.008	*p* < 0.01
*Stressful life events → eating out → obesity at baseline→ obesity at follow-up*	0.002	0.001	0.001–0.004	*p* < 0.05

^a^ Bias-corrected percentile method was presented based on 5000 bootstraps samples.

## Data Availability

The datasets used and/or analyzed in this study are available from the corresponding author S.X. upon reasonable request.

## Supplementary Materials

The following supporting information can be downloaded at: https://www.mdpi.com/article/10.3390/nu15112637/s1. Figure S1. Inclusion process for the participants. Table S1. Characteristics of different unhealthy eating behavior. Table S2. Baseline characteristics of different type stressful life events.
