# Supplementary material for: Stressful Life Events, Unhealthy Eating Behaviors and Obesity among Chinese Government Employees: A Follow-Up Study
Source: Nutrients. 2023 Jun 5;15(11):2637. doi: 10.3390/nu15112637 (PMC10255864; doi:10.3390/nu15112637)
Supplement: Supplementary file 1 [file nutrients-15-02637-s001.zip › nutrients-2418754-supplementary.pdf]

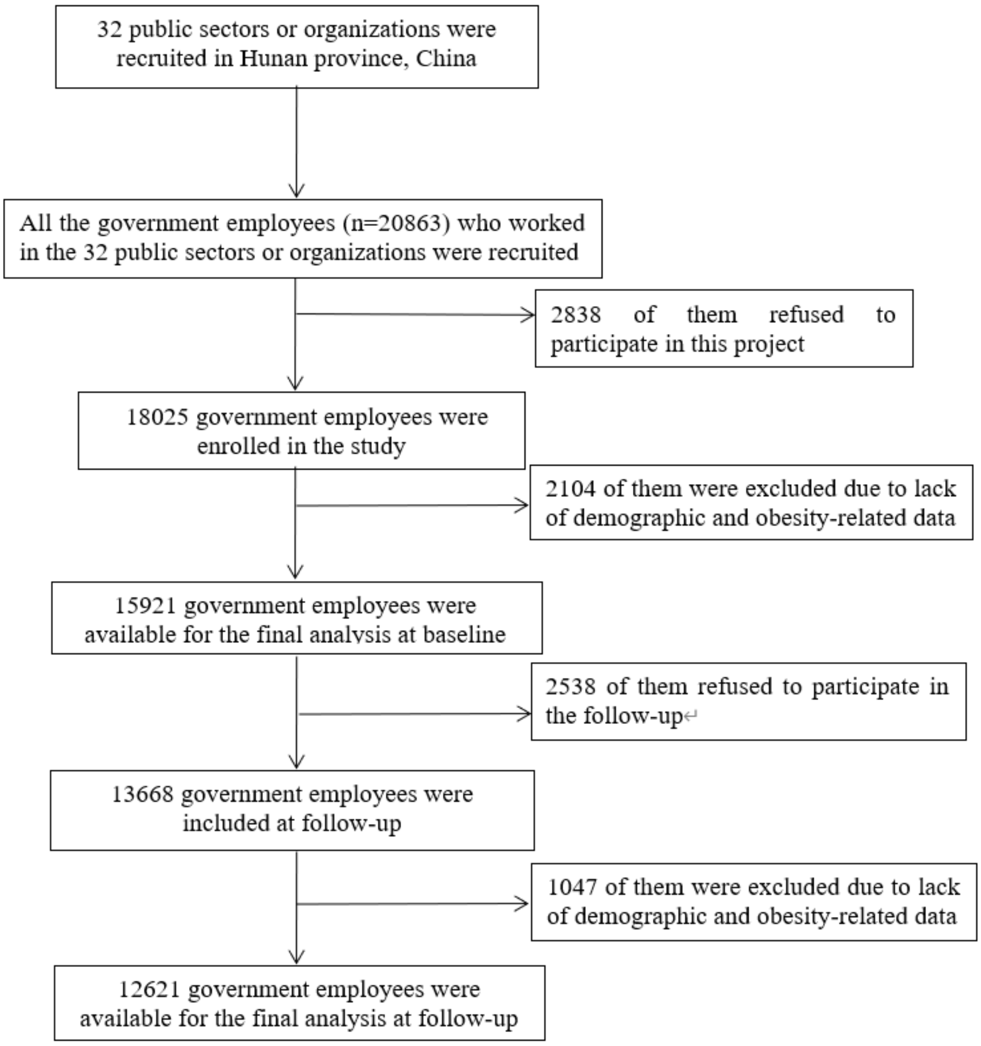

Figure S1 Inclusion process for the participants

Table S1. characteristics of different unhealthy eating behavior

	Baseline(n=15,921)		Follow-up (n=12,642)	
Irregular meal timing	n	%	n	%
No	8632	54.2%	6839	54.2%
Sometimes	5766	36.2%	4572	36.2%
Often	1523	9.6%	1210	9.6%
Overeating at each mealtime				
No	14952	93.9%	11909	94.4%
Sometimes	969	6.1%	712	5.6%
Often	-	-	-	-
Eating before going to bed at night				
No	8353	52.5%	6916	54.8%
Sometimes	7021	44.1%	5304	42.0%
Often	547	3.4%	401	3.2%
Eating out				
No	13936	87.5%	11184	88.6%
Sometimes	1732	10.9%	1258	10.0%
Often	253	1.6%	179	1.4%

Table S2. Baseline characteristics of different type stressful life events

Type of life events	Item	N	%
Events related to family & marriage		7115	56.3
	In love or engagement	1569	12.4
	Broken love	599	4.7
	Get married	719	5.7
	Pregnancy or wife pregnancy	1863	14.7
	Miscarriage by yourself or wife	586	4.6
	Add new members to the family	1711	13.5
	Discord with spouse's parents	1433	11.3
	Bad relationship between spouse	1250	9.9
	Husband and wife separated due to discord	410	3.2
	Unsatisfied sex life	1009	8.0
	Separation from spouse due to work demands	1300	10.3
	Spouse has an affair	119	0.9
	Marital relationship becomes better	767	6.1
	Violation of one child policy	37	0.3
	Yourself or your spouse undergo sterilization	102	0.8
	Death of spouse	43	0.3
	Divorce	152	1.2
	Failure of children's advancement or employment	154	1.2
	Difficulties in child discipline	1072	8.5
	Children leave home for a long time	314	2.5
	Parental discord	507	4.0
	Family member seriously ill/ injured	742	5.9
	Death of family member	529	4.2

	Go abroad for the first time	308	2.4
	Friends seriously ill/ injured	226	1.8
	Friend died	223	1.8
Events related to work		5255	41.6
	Unemployed	131	1.0
	Start working	602	4.8
	Deduction of bonuses and fines	867	6.9
	Outstanding personal achievement	449	4.1
	Promotion	961	7.6
	Not satisfied with the current job	1874	14.8
	Work stress	3600	28.5
	Poor relationship with superiors	670	5.3
	Discord with colleagues or neighbors	281	2.2
	Retired or did not arrange a specific job	97	0.8
	Examination failed	54	0.4
Events related to economic problems		2625	20.8
	Family financial difficulties	921	7.3
	In debt	1361	10.8
	The economic situation has improved significantly	1110	8.8
	Housing shortage	482	3.8
Events related to accidents and legal disputes		1378	10.9
	Misunderstood, blamed, false accusation, argument	1024	8.1
	Theft, property damage	252	2.0
	Unexpected frights, accidents, natural disasters	202	1.6
	Intervene in civil legal disputes	182	1.4
	Detained, tried	41	0.3
Events related to health		1109	8.8
	Lifestyle changes	980	7.8
	Seriously ill/injured	181	1.4
